# Supplementary material for: Bedaquiline reprograms central metabolism to reveal glycolytic vulnerability in Mycobacterium tuberculosis
Source: Nat Commun. 2020 Nov 30;11:6092. doi: 10.1038/s41467-020-19959-4 (PMC7705017; doi:10.1038/s41467-020-19959-4)
Supplement: Supplementary file 1 — Supplementary Information [file 41467_2020_19959_MOESM1_ESM.pdf]

## SUPPLEMENTARY INFORMATION

# Bedaquiline reprograms central metabolism to reveal glycolytic vulnerability in *Mycobacterium tuberculosis*

Jared S. Mackenzie[1], Dirk A. Lamprecht[2], Rukaya Asmal[1], John H. Adamson[1], Khushboo Borah[3], Dany J.V. Beste[3], Bei Shi Lee[4], Kevin Pethe[4,5], Simon Rousseau[6], Inna Krieger[6], James C. Sacchettini[6], Joel N. Glasgow[7] and Adrie J. C. Steyn[1,7,8*]

[1]Africa Health Research Institute, Durban 4001, South Africa.
[2]Janssen Pharmaceutica, Global Public Health, Turnhoutseweg 30, 2340 Beerse, Belgium.
[3]Faculty of Health and Medical Sciences, University of Surrey, Guildford, UK
[4] School of Biological Sciences, Nanyang Technological University, Singapore, Singapore.
[5]Lee Kong Chian School of Medicine, Nanyang Technological University, Singapore, Singapore.
[6]Texas A&M University, Department of Biochemistry and Biophysics, College Station, TX, USA
[7]Department of Microbiology, University of Alabama at Birmingham, Birmingham, AL, USA.
[8]Center for AIDS Research and Center for Free Radical Biology, University of Alabama at Birmingham, Birmingham, AL USA.

*Corresponding Author
Adrie JC Steyn, Ph.D.
asteyn@uab.edu
+27-31-260-4715

## Supplementary Figures

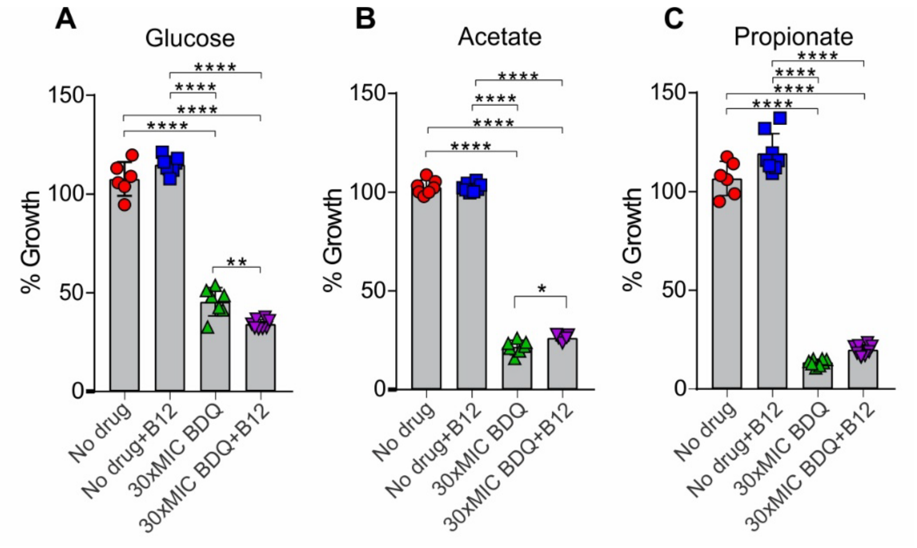


**Supplementary Figure 1: Vitamin $B_{12}$ does not reduce susceptibility of *Mtb* to BDQ.** A Microplate Alamar Blue Assay (MABA) was used to determine the effect of vitamin B12 on BDQ-mediated inhibition of *Mtb* growth. The potential effect of vitamin B12 (B12) was investigated using three carbon sources: (**A**) glucose, (**B**) acetate and (**C**) propionate. Two independent experiments were performed in separate 96 well plates, and pooled data (n= 5-8) from both experiments are shown. Error bar indicates SEM. A One-Way ANOVA and a Tukey multiple comparisons test were used for statistical analysis. n = 6. *$p<0.05$, **$p<0.01$, ****$p<0.0001$.

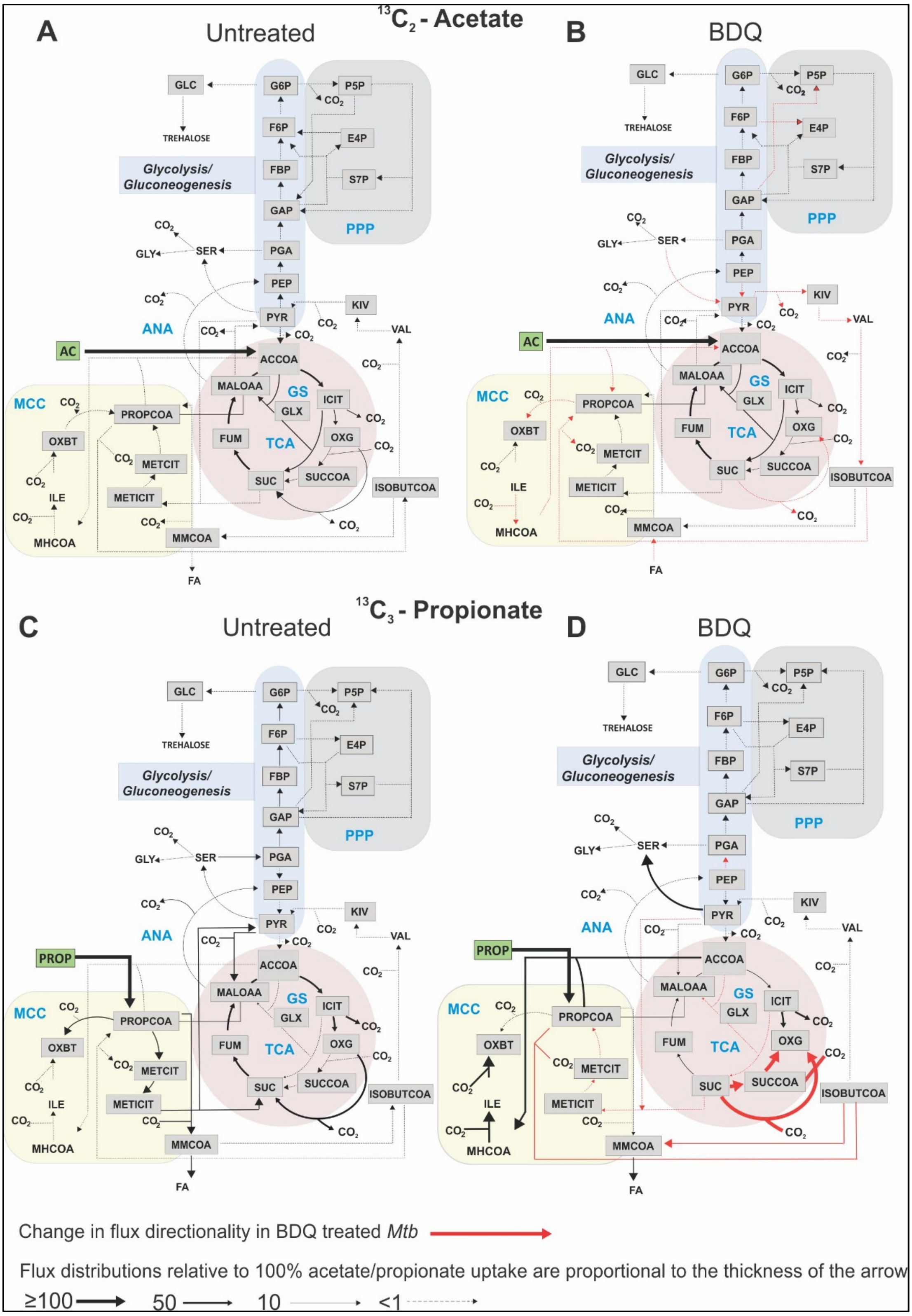


**Supplementary Figure 2: BDQ treatment induces a change in flux directionality.** Flux maps of untreated and BDQ-treated *Mtb* growing on (A and B) acetate and (C and D) propionate as the primary carbon source. The fluxes measured were proportional to the acetate/propionate uptake flux set to 100. Comparison between untreated and treated conditions identifies the change in flux direction upon BDQ treatment and are highlighted in red. G6P – glucose 6 phosphate; F6P – fructose 6 phosphate; FBP – fructose 1,6

bisphosphate; GAP – glyceraldehyde 3 phosphate; P5P -pentose 5 phosphate; E4P – erythsorse 4 phosphate; S7P – sedoheptulose 7 phosphate; SER – serine; PGA – 3 phosphglyceric acid; PEP – phosphoenolpyruvate; PYR – pyruvate; KIV – 2 ketoisovalerate; VAL – valine; ACCOA – acetyl CoA; ICIT – isocitrate; OXG – alpha ketoglutarate; SUCCOA – succinyl CoA; ISOBUTCOA – isobutyrylCoA; SUC – succinate; FUM – fumarate; MALOAA – malate and oxaloacetate; PROPCOA – Propionyl CoA; METCIT – methylcitrate; METICIT – methylisocitrate; ILE – isoleucine; FA – fatty acids.

**Supplementary Table 1:** Network model of *Mtb* central metabolism

| Reaction | Stoichiometry |
|---|---|
| Glycolysis | F6P ↔ G6P |
| Glycolysis | FBP ↔ F6P |
| Glycolysis | GAP + GAP ↔ FBP |
| Glycolysis | GAP ↔ PGA |
| Glycolysis | PGA ↔ PEP |
| Glycolysis PYK/PPDK | PEP ↔ PYR |
| PPP | G6P → P5P + $CO_2$ |
| PPP | GAP + F6P ↔ P5P + E4P |
| PPP | S7P + GAP ↔ P5P + P5P |
| PPP | E4P + F6P ↔ GAP + S7P |
| Glycolysis PDH | PYR → ACCOA + $CO_2$ |
| TCA | MALOAA + ACCOA → ICIT |
| TCA | ICIT ↔ OXG + $CO_2$ |
| TCA | OXG ↔ SUCCOA + $CO_2$<br>OXG ↔ SSA + $CO_2$ |
| TCA | SUCCOA ↔ SUC<br>SSA ↔ SUC |
| TCA | SUC ↔ FUM |
| TCA | FUM ↔ MALOAA |
| Glyoxylate shunt | ICIT → GLX + SUC |
| Glyoxylate shunt | GLX + ACCOA → MALOAA |
| Anaplerosis | PEP + $CO_2$ ↔ MALOAA |
| Anaplerosis | PYR + $CO_2$ ↔ MALOAA |
| Methylcitrate cycle | PROPCOA + MALOAA ↔ METICIT |
| Methylcitrate cycle | METCIT ↔ METICIT |
| Methylcitrate cycle | METICIT ↔ SUC + PYR |
| Alanine biosynthesis | PYR → ALA |
| Valine biosynthesis | PYR + PYR → KIV + $CO_2$ |
| Valine biosynthesis | KIV → VAL |
| Valine degradation | OXG + VAL ↔ ISOBUTCOA + CO2 + GLU |
| Valine degradation | ISOBUTCOA ↔ MMCOA |
| Valine degradation | ISOBUTCOA ↔ CO2 + PROPCOA |
| Leucine biosynthesis | KIV + ACCOA → LEU + $CO_2$ |
| Serine biosynthesis | GAP → SER |
| Serine degradation | PYR ↔ SER |
| Glycine biosynthesis | SER → GLY + $CO_2$ |
| Histidine biosynthesis | P5P → HIS |
| Aspartate biosynthesis | MALOAA → ASP |
| Threonine biosynthesis | ASP → THR |
| Threonine degradation | THR ↔ OXBT |
| Threonine degradation | OXBT ↔ PROPCOA + CO2 |
| Threonine degradation | OXBT + PYR + GLU ↔ ILE + CO2 + OXG |
| Methionine biosynthesis | ASP → MET |
| Lysine biosynthesis | PYR + ASP → LYS + $CO_2$ |
| Isoleucine biosynthesis | THR + PYR → ILEU + $CO_2$ |
| Isoleucine degradation | ILE + OXG ↔ MHTPP + CO2 + GLU |
| Isoleucine degradation | MHTPP ↔ PROPCOA + ACCOA |
| Isoleucine degradation | PROPCOA + CO2 ↔ MMCOA |
| Glutamate biosynthesis | OXG → GLU |
| Proline biosynthesis | GLU → PRO |
| Ornithine biosynthesis | GLU → ORN |
| Chorismate biosynthesis | E4P + PEP → CHO |
| Phenylalanine biosynthesis | CHO + PEP → PHE + $CO_2$ |
| Tyrosine biosynthesis | CHO + PEP → TYR + $CO_2$ |

Abbreviations: PYK- pyruvate kinase; PPDK-pyruvate dikinase; PDH-pyruvate dehydrogenase; TCA-tricarboxylic acid cycle; SSA-succinylsemialdehyde; F6P-fructose6phosphate; G6P-glucose6phosphate; GAP-glyceraldehyde3phosphate; PGA-phosphoglyceric acid; PYR-pyruvate; PEP-phosphoenolpyruvate; P5P-pentose5phosphate; S7P-sedoheptoluse7phosphate; E4P-erythrose4phosphate; AACOA-acetylecoenzymeA; MMCOA-methylmalonylcoenzymeA; PROPCOA-propionylcoenzymeA; OXBT-oxobutanoate; FUM-fumarate; MALOAA-malateoxaloacetate; SUC-succinate; SUCCOA-succinylcoenzymeA; OXG-2oxoglutarate; CIT-citrate; ICIT-isocitrate; METCIT-methylcitrate; METICIT-methylisocitrate; GLX-glyoxylate; ALA-alanine;VAL-valine; KIV-ketoisovalarate; LEU-leucine; ILE-Isoleucine; SER-serine; GLY-glycine; THR-threonine; MET-methionine; LYS-lysine; TYR-tyrosine; PHE-phenylalanine; CHO-chorismate; ASP-aspartate; GLU-glutamate; ISOBUTCOA-isobutyrylcoenzymeA; MHTPP-Methylhydroxybutyl ThPP; ORN-ornithine; PRO-proline; HIS-histidine.
